# Acute-Stress Biomarkers in Three Octopodidae Species After Bottom Trawling

**DOI:** 10.3389/fphys.2019.00784

**Published:** 2019-06-25

**Authors:** Cristina Barragán-Méndez, Ignacio Sobrino, Adrián Marín-Rincón, Sergio Fernández-Boo, Benjamin Costas, Juan Miguel Mancera, Ignacio Ruiz-Jarabo

**Affiliations:** ^1^Department of Biology, Faculty of Marine and Environmental Sciences, Campus de Excelencia Internacional del Mar, Universidad de Cádiz, Cádiz, Spain; ^2^Instituto Español de Oceanografía, Centro Oceanográfico de Cádiz, Cádiz, Spain; ^3^Centro Interdisciplinar de Investigação Marinha e Ambiental, Universidade do Porto, Matosinhos, Portugal; ^4^Instituto de Ciências Biomédicas Abel Salazar, Universidade do Porto, Porto, Portugal

**Keywords:** acute stress, bottom trawling, *Eledone*, Octopodidae, *Octopus*, physiological recovery

## Abstract

Several Octopodidae species have a great potential for the diversification of worldwide aquaculture. Unfortunately, the lack of stress-related biomarkers in this taxon results an obstacle for its maintenance in conditions where animal welfare is of paramount relevance. In this study, we made a first approach to uncover physiological responses related to fishing capture in *Eledone moschata*, *Eledone cirrhosa*, and *Octopus vulgaris*. Captured octopus from all three species were individually maintained in an aquaculture system onboard of oceanographic vessel in south-western waters of Europe. Haemolymph plasma and muscle were collected in animals at the moment of capture, and recovery was evaluated along a time-course of 48 h in *Eledone* spp., and 24 h for *O. vulgaris*. Survival rates of these species captured in spring and autumn were evaluated. Physiological parameters such as plasma pH, total CO_2_, peroxidase activity, lysozyme, hemocyanin, proteases, pro-phenoloxidase, anti-proteases, free amino acids, lactate and glucose levels, as well as muscle water percentage, free amino acids, lactate, glycogen and glucose values were analyzed. The immune system appears to be compromised in these species due to capture processes, while energy metabolites were mobilized to face the acute-stress situation, but recovery of all described parameters occurs within the first 24 h after capture. Moreover, this situation exerts hydric balance changes, as observed in the muscle water, being these responses depending on the species assessed. In conclusion, three Octopodidae species from south-western waters of Europe have been evaluated for stress-related biomarkers resulting in differentiated mechanisms between species. This study may pave the way to further study the physiology of stress in adult octopuses and develop new methodologies for their growth in aquaculture conditions.

## Introduction

Cephalopods are of interest for human consumption and their fisheries constituted 6.4% of total world trade of fish products in 2016, and are amongst the most captured species (in tons) in marine fisheries (FAO, 2018). Wild octopus are caught every year, reaching 350,000 tons with a trade value of 1.5 billion dollars (Mouritsen and Styrbaek, 2018), being China and Morocco the largest exporters of octopus, while Japan, the United States and Spain are the most important consumer markets (FAO, 2018).

Landings of octopus in Europe consist exclusively of three Octopodidae species: musky octopus (*Eledone moschata* Lamarck, 1798), horned octopus (*Eledone cirrhosa* Lamarck, 1798) and common octopus (*Octopus vulgaris* Cuvier, 1797). The latter species dominates European catches and landings, and is taken in greater numbers in southern waters (Pierce et al., 2010). In south Atlantic waters of Europe (Gulf of Cadiz, Spain) all three octopus species are captured by an artisanal and bottom trawling fleet (Sobrino et al., 2011). While *O. vulgaris* is the target species accounting for more than 87% of total Spanish octopus catches due to its high economically value in market, both *Eledone* spp. are routinely discarded after trawling due to their low market prices (Jereb et al., 2015). Bathymetric differences exist between these species, with *O. vulgaris* inhabiting depths from 0 to 200 m, *E. moschata* could be captured between 15 to 200 m depth, and *E. cirrhosa* occurs at deeper waters, between 50 and 300 m, or more (Pierce et al., 2010).

Octopus landings have declined fairly consistently since the mid-1980s (Jereb et al., 2015). Some pressure has been also generated on these species after the new Common Fisheries Policy in Europe, as it points out at a compulsory landing obligation for those species regulated by quotas or minimum sizes, including captured juvenile octopus (Regulation 1380/2013/UE). However, as reported in Article 13 of the regulation, captured species could be released back into the sea if robust scientific evidences indicated high survival rates and physiological recovery. Moreover, yearly variations on octopus captures due to environmental processes such as rainfalls, river discharges, and oceanographic currents are of great importance (Sobrino et al., 2002; Sánchez et al., 2004; Sobrino et al., 2011; Otero et al., 2016; Roura et al., 2019), highlighting aquaculture as a mandatory future to provide an increasing market supply of these species.

It is known that stress is a physiological response aimed to maintain the basal homeostatic levels of an organism (Chrousos, 2009). In vertebrates, these physiological responses have been broadly grouped as primary, secondary and tertiary (Barton, 2002), but knowledge on cephalopods is scarce. Primary stress responses include the release of neuroendocrine messengers such as catecholamines in cephalopods (Malham et al., 2002) and vertebrates (Reid et al., 1998), amongst other hormones (Schreck et al., 2016). Secondary stress responses are defined by the actions promoted by these hormones, including changes in the management of energy resources and the immune system (Costas et al., 2011; Schreck et al., 2016). If the stressful situation extends over time it can lead to exhaustion of the energy reserves, depression of the immune system, impairment of the behavior and reproduction, and eventually death of the animal (Wedemeyer et al., 1990; Schreck et al., 2016).

It was described that intermediary metabolism of cephalopods relies in amino acids and carbohydrates as a primary source of energy (Aguila et al., 2007; Lamarre et al., 2016; Speers-Roesch et al., 2016; Morales et al., 2017). In this sense, the muscle of molluscs is a source of proteins and carbohydrates that may be used as catabolic substrates when necessary (Lee et al., 2015). Thus, muscle glycogen seems to be an important source of glucose to be oxidized through glycolytic pathways (Storey and Storey, 1983), producing lactate under anaerobic circumstances (Gladden, 2004).

After acute-stress situations, sustained anaerobiosis results in a net accumulation of protons and metabolic CO_2_ and hence, plasma acidosis (Hochachka et al., 1983). Changes in pH and temperature regulate oxygen transport in the haemolymph (Oellermann et al., 2015a, b). Hemocyanin (Hc), the respiratory pigment in cephalopods, is dissolved in the haemolymph and accounts for 98% of total proteins present in octopus blood (Aguila et al., 2007). It should be noticed that Hc exhibits phenoloxidase (PO) activity in cephalopods and crustaceans, which is involved in the innate defense mechanism (Adachi et al., 2003; Lacoue-Labarthe et al., 2009), and has shown to be affected by repeated sampling procedures in *E. cirrhosa* (Malham et al., 1998a) and infections in *O. vulgaris* (Castellanos-Martinez et al., 2014). The pro-PO activation is produced by proteases (Decker and Jaenicke, 2004). The immune system in cephalopods is poorly known to date, and lacks an adaptive immune response, but shows a good and efficient innate immune system composed of cellular and humoral (dissolved proteins in plasma) defense factors (Castellanos-Martinez et al., 2014). Thus, lysozyme activity appears to act non-specifically against a wide range of invaders in *E. cirrhosa* (Malham et al., 1998b), peroxidases possess antimicrobial activity that eliminates H_2_O_2_, maintaining the redox balance of the immune system (Rodríguez et al., 2003), antiproteases protect against bacterial proteases as well as endogenous proteases released by host cells (Malham and Runham, 1998; Malham et al., 1998b), while proteases like anti-trypsin show anti-inflammatory activity (Guttman et al., 2015).

The aim of this study was to evaluate acute-stress responses in three Octopodidae species (*E. moschata*, *E. cirrhosa*, and *O. vulgaris*). As fisheries processes were described as a source of acute-stress for the organisms (Lund et al., 2009; Gallagher et al., 2014; Veldhuizen et al., 2018), animals were captured by bottom trawling in the Gulf of Cadiz (south western waters of Europe) and allow to recover in onboard aquaria. Survival rates of these species were also evaluated altogether with physiological recovery responses. Results from this study may serve to improve the aquaculture of these species.

## Materials and Methods

### Geographic Location, Vessel and Tows Characteristics

**FIGURE 1:**
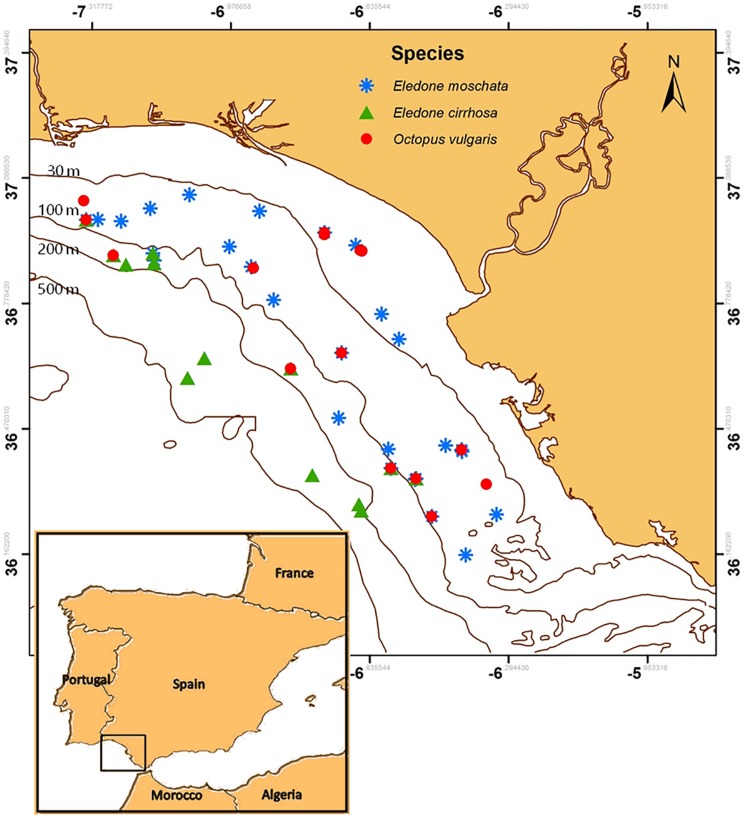
Sampled area off the Gulf of Cadiz (south-western Atlantic waters of Europe, Spain). Blue asterisks, green triangles, and red circles indicate sampling points of *Eledone moschata*, *Eledone cirrhosa*, and *Octopus vulgaris*, respectively. Bathimetric lines and their depths (m) are indicated.

Octopus were captured by bottom trawling aboard the O/V “Miguel Oliver” (length: 70 m; engine power: 2x 1000 kw) during three different trawling surveys off the Gulf of Cadiz (south-western waters of Europe, Spain) in spring (March 2017 and 2018), and autumn (November 2017) (Figure 1). International standards were used during fishing processes, including 1 h of trawling at a constant depth (ANON, 2015). Temperature and salinity values were collected in each haul by a conductance-temperature-density probe (CTD) placed in the net.

### Animals Employed

Three Octopodidae species were studied to reinforce the results from an inter-specific point of view: *E. moschata*, *E. cirrhosa*, and *O. vulgaris*. Captures in this area depend on yearly variations (Sánchez et al., 2004; Sobrino et al., 2011) and were not constant along this study (Table 1). Depth of capture, as well as environmental temperature and salinity at the bottom, at the surface and in the recovery aquaria is shown in Table 1. Animals of both sexes were randomly selected aboard the vessel. *E. moschata* and *E. cirrhosa* weight was 197 ± 32 g and 117 ± 1 g, respectively. As *O. vulgaris* is an economically important species, with a minimum commercial size of 1 kg, this study was conducted with animals below that size, weighting 463 ± 26 g. Animals were kept and handled following the guidelines for experimental procedures in animal research from the Ethics and Animal Welfare Committee of the University of Cadiz, according to the Spanish (RD53/2013) and European Union (2010/63/UE) legislation. All experiments have been carried out under a special permit of scientific fishing granted to the Spanish Institute of Oceanography, and approved by the Spanish General Secretariat of Fisheries (project SUREDEPAR, Fundación Biodiversidad, Ministry for the Ecological Transition, Spain).

**TABLE 1:** Environmental conditions of the experiments conducted in this study, including parameters of the fishing area (bottom), and temperature in the aquariums (tanks) and in the air of the fishing deck (air) with *E. moschata*, *Eledone Cirrhosa*, and *Octopus vulgaris* captured during bottom-trawling in the Gulf of Cadiz (Spain).

**Species**	**Season year**	**Exp.**	**Depth (m)**	**Salinity (psu)**	**Temperature (°C)**		
					**Bottom**	**Tanks**	**Air**
*E. moschata*	Spring 2017	R	76 ± 18	36.2 ± 0.1	14.0 ± 0.5	16.5 ± 0.1	18.6 ± 0.7
	Autumn 2017	S	78 ± 9	36.2 ± 0.0	16.6 ± 0.5	21.3 ± 0.3	22.6 ± 0.7
	Spring 2018	S	71 ± 10	36.3 ± 0.0	15.4 ± 0.1	17.4 ± 0.1	18.6 ± 0.1
*E. cirrhosa*	Spring 2017	R	290 ± 77	36.2 ± 0.1	13.9 ± 0.2	16.5 ± 0.1	18.6 ± 0.7
	Spring 2018	S	208 ± 49	36.3 ± 0.0	15.2 ± 0.2	17.4 ± 0.1	18.6 ± 0.1
*O. vulgaris*	Autumn 2017	R, S	21 ± 2	36.5 ± 0.0	21.0 ± 0.2	21.3 ± 0.3	22.6 ± 0.7
	Spring 2018	S	87 ± 12	36.3 ± 0.0	15.4 ± 0.1	17.4 ± 0.1	18.6 ± 0.1

*“Exp.” indicates the experiments during that season, including time-course recovery studies (R), and evaluation of the survival rates (S). Data are represented as mean ± SEM.*

### Recovery Aquaria Onboard

After bottom trawling, no more than 10 animals per species and trawl were introduced into onboard aquaria and allow to recover. A portable aquaculture system was specially designed for this purpose. This system consists of 30 independent aquariums, painted in black with an upper light entrance, of 5 L each with a flow-throw system of seawater collected from the surface of the ocean during navigation. The system also has a charcoal filter and a protein skimmer to remove possible contaminants and/or dissolved nitrogenous molecules. Animals suffered around 65 min of air exposure (*E. moschata* 68 ± 2 min, *E. cirrhosa* 63 ± 7 min, and *O. vulgaris* 64 ± 2 min, without statistical differences between species), mimicking commercial fisheries in the Gulf of Cadiz, Spain (personal observation), in a lower fishing deck where environmental conditions include high environmental humidity, no sun radiation, low room irradiance, and constant temperatures below 23°C (see Table 1) before being introduced into the aquaria. Animals were fasted during experiments onboard. Dissolved oxygen was maintained above 80% saturation (>6.5 mg O_2_ L^–1^) by means of external aeration of the collected seawater.

### Physiological Recovery Curve

Animals were sampled (see description in see section “Sampling”) at times 0 h (immediately after capture and after air exposure) and after a few hours of being introduced into the aquaria. We aimed at the evaluation of the physiological recovery by conducting a time-course survey, assuming that acute-stress responses have been overcome when the physiological variables analyzed reach stable values over time, as described before in crustaceans (Barragán-Méndez et al., unpublished), elasmobranches (Barragán-Méndez et al., 2019), and teleosts (Skrzynska et al., 2018). In order to carry out this objective *E. moschata* (*n* = 55) and *E. cirrhosa* (*n* = 36) captured during 4 independent trawls for each species in March 2017 were randomly introduced into the recovery aquaria. A group of randomly selected animals was sacrificed before being introduced into the aquaria and samples were taken, constituting time 0 h (*n* = 18 *E. moschata* and *n* = 12 *E. cirrhosa*). Those animals introduced into the aquaria were maintained for 24 h (*n* = 18 *E. moschata* and *n* = 12 *E. cirrhosa*) and 48 h (*n* = 19 *E. moschata* and *n* = 12 *E. cirrhosa*) before being sampled. No *O. vulgaris* were captured in March 2017 but in November 2017 (*n* = 44 animals captured in 7 independent trawls). As both *Eledone* spp. appeared to be physiologically stable after 24 h (all analyzed parameters collected in March 2017 shown significantly similar values between times 24 and 48 h for each species, see section “Results”), and physiological recovery in crustaceans (Barragán-Méndez et al., unpublished) and teleost fish (Costas et al., 2011) occurs within the first 4 to 6 h, *O. vulgaris* were sampled at time 0 h (*n* = 15), 6 h (*n* = 15), and 24 h (*n* = 14) after recovery in the aquaria. A maximum of five animals per species and trawl were sampled at each time to reinforce independence of the results.

### Survival Rates in Spring and Autumn

In order to study differences due to seasonality in the octopus’ survival rates after capture, *E. moschata* were collected in autumn 2017 and in spring 2018 (from 10 and 13 hauls, respectively), *O. vulgaris* were collected in autumn 2017 and in spring 2018 (from 7 and 12 hauls, respectively), and *E. cirrhosa* were only captured in spring 2017 (11 hauls). After the triage process in the lower fishing deck aboard, a maximum of 5 octopus per species and trawl were introduced into the recovery aquaria and survival rates were evaluated 24 h later, as it was stated that giant Pacific octopus showed no retarded mortality after 24 h recovery (Conners and Levine, 2017). Animals were monitored every other hour (from 8 am to 1 am) during the experiments. Octopuses were considered alive if they show signs of breath, and responded to physical stimulation by moving their bodies when the observer opened their aquaria. Dead animals show a clear pale body colouration, a lack of body movements and rigidness. Dead animals were immediately removed from their aquaria.

### Physiological Recovery in Spring and Autumn

In order to evaluate possible physiological differences between animals captured in spring and autumn, those animals employed in the survival rate evaluation were sampled. Haemolymph and muscle samples were collected at time 0 h (after circa 65 min of air exposure), and 24 h after recovery.

### Sampling

Animals were anesthetized by decreasing water temperature to circa 4°C (by covering the aquariums with ice for 5 min, without physical disturbance of the animals) and addition of 2.5% ethanol for 2 min after that, conforming the principles of Directive 2010/63/EU, and following described procedures (Malham et al., 2002; Estefanell et al., 2011; Sancho et al., 2015). Haemolymph samples were taken with sterile 1 mL 25 G syringes (circa 300 μL per animal) from the principal heart as described before (Aguila et al., 2007). Euthanasia was confirmed by severing the brain between the eyes. Muscle samples were collected from the third left arm. All samples were taken quickly (in less than 1 min per animal) to avoid an additional stress due to handing (Malham et al., 2002; Lawrence et al., 2018). As octopuses were maintained in individual aquaria, with darkened walls, the sampling process did not affect remaining animals in the aquarium (i.e., there were no disturbances due to visual contact, noise, or chemical distress). The soluble fraction of the haemolymph (referred to plasma hereafter, unless it could contain cytoplasmic components of the haemocytes) was obtained after centrifugation (10,000 × *g*, 4 min). All samples were immediately frozen at −20°C (by employing a refrigerated mixture that immediately freeze the samples). The samples were maintained at −20°C for less than 9 days, transferred to the Department of Biology (University of Cadiz, Spain) in dry ice and then maintained at −80°C.

### Analysis of Plasma and Muscle Parameters

#### Plasma pH and TCO_2_

Plasma pH was measured immediately after centrifugation with a mini-electrode (HI1083B, Hanna Instruments, Rhode Island, United States), and a 15 μL sample was collected for immediate analysis of total CO_2_ (TCO_2_) by means of an infra-red gas analyser (IRGA, S151, Qubit systems, Kingston, ON, Canada). The protocol for TCO_2_ analysis was as follows: 15 μL of plasma were introduced into a 3.0 mL exetainer vial (Labco, United Kingdom) under a CO_2_/water-free atmosphere (air pumped throw a 2 m length and a diameter of 18 mm tube filled with ½ silica gel and ½ soda lime) and hermetically closed. 100 μL 0.1 N HCl were injected into the vial and vigorously mixed with the sample. The needle of an empty 5 mL syringe was introduced into the vial followed by the introduction of the needle of a 5 mL syringe full of CO_2_/water-free air. Air inside the vial was mixed by means of the two 5 mL syringes. Finally, 5 mL of that mixtured air was introduced into the IRGA and the concentration (in ppm) of CO_2_ was analyzed. A standard curve was done with serial dilutions of NaHCO_3_ diluted in distilled water (0 mM HCO_3_^–^).

#### Plasma Photographs *in vivo*

Haemolymph was photographed when circulating inside the blood system and while sampling as a proxy to estimate oxygen saturation rates between experimental groups. Due to the high amount of hemocyanin (Hc) in the plasma, haemolymph turns blue when Hc is oxidized (due to the cupper ions of the Hc molecule), and translucent when reduced. We aimed at grossly differentiate colored haemolymph between sampling groups.

#### Plasma Energy Metabolites

Plasma glucose and lactate levels were analyzed using commercial kits from Spinreact (St. Esteve de Bas, Girona, Spain) adapted for 96-well microplates. Total α-amino acid levels were assessed colorimetrically using the ninhydrin method of Moore (Moore, 1968) adapted for 96-well microplates. Plasma total proteins were determined using the Pierce BCA protein assay kit (Thermo Fisher Scientific) according to manufacturer recommendations.

#### Plasma Immune Status

Plasma hemocyanin concentration was measured spectrophotometry as previously described for octopuses (Aguila et al., 2007; Roumbedakis et al., 2017).

Plasma lysozyme activity was measured as described (Swain et al., 2007): 20 μL of sample and 180 μL of a solution of *Micrococcus lysodeikticus* (N3770, Sigma-Aldrich; 0.2 mg mL^–1^, 0.04 M sodium phosphate buffer, pH 6.2) were added into a 96-well microplate. Blanks for each sample were done with 20 μL of the sample and 180 μL of sodium phosphate buffer. Reaction proceeds for 20 min at 37°C and afterward absorbance was measured at 450 nm. A standard curve was done with lyophilized hen egg white lysozyme (L6876, Sigma-Aldrich) serially diluted in Na_2_HPO_4_ buffer.

Peroxidase activity was measured as described (Quade and Roth, 1997), with some modifications: 15 μL plasma in duplicate were diluted in 135 μL of HBSS without Ca^2+^/Mg^2+^ (H6648, Sigma-Aldrich) in a flat-bottomed 96-well plate, followed by the addition of 50 μL 10 mM TMB (T8768, Sigma-Aldrich) and 50 μL 5 mM H_2_O_2_. After 2 min the reaction was stopped with 50 μL 2 M H_2_SO_4_. Blank was done with 150 μL HBSS. Optical density was read at 450 nm. Peroxidase activity (U mL^–1^) was determined defining 1 unit as that which produces an absorbance change of 1 OD.

Total PO-like activity activity was measured spectrophotometrically using L-DOPA (L-3,4-dihydroxyphenylalanine) as substrate, and trypsin (Sigma) as activator following described methodologies (Ji et al., 2009) with some modifications. Briefly, 15 μL plasma were incubated for 30 min at 25°C with 100 μL trypsin (1 mg mL^–1^), with further addition of 100 μL L-DOPA (3 mg mL^–1^) and absorbance measurements every 5 min at 490 nm in a SynergyHT microplate reader. Units of PO-like activity were calculated using Lambert–Beer law taking the gradient of slope of each sample and molar extinction coefficient of the L-DOPA (3700 M^–1^ cm^–1^).

Protease activity was quantified using the azocasein hydrolysis assay according to the method of Ross et al. (2000) with some modifications. Briefly, protease activity was assayed in 10 μL plasma with 60 μL PBS and 125 μL 2% azocasein in 100 mM ammonium bicarbonate buffer. Samples were incubated for 24 h at 30°C. The reaction was stopped by adding 250 μL 10% trichloro acetic acid and the mixture centrifuged (10,000 × *g*, 10 min). 100 μL of the supernatant was mixed with 100 μL 1 N NaOH and optical density read at 450 nm. Trypsin was employed as standard.

Total antiprotease activity was determined by the ability of plasma to inhibit trypsin activity according to Ellis (1990) and modifications by Machado et al. (2015). Briefly, 10 μL of plasma were incubated with the same volume of a trypsin solution (5 mg mL^–1^ in NaHCO_3_, 5 mg mL^–1^, pH 8.3) for 10 min at 22°C in polystyrene microtubes. To the incubation mixture, 100 μL of phosphate buffer (NaH_2_PO_4_, 13.9 mg mL^–1^, pH 7.0), and 125 μL of azocasein (20 mg mL^–1^ in NaHCO_3_, 5 mg mL^–1^, pH 8.3) were added and incubated for 1 h at 22°C. Finally, 250 μL of trichloroacetic acid were added to each microtube and incubated for 30 min at 22°C. The mixture was centrifuged at 10,000 × *g* for 5 min at room temperature. Afterward, 100 μL of the supernatant was transferred to a 96 well-plate that previously contained 100 μL of NaOH (40 mg mL^–1^) per well. The OD was read at 450 nm in a SynergyHT microplate reader. Phosphate buffer in place of plasma and trypsin served as blank whereas the reference sample was phosphate buffer in place of plasma. The percentage inhibition of trypsin activity compared to the reference sample was calculated. All analyses were conducted in duplicates.

#### Muscle Energy Metabolites and Water Content

Frozen muscle was finely minced on an ice-cooled Petri dish and homogenized by ultrasonic disruption in 7.5 volumes ice-cold 0.6 N perchloric acid, neutralized using 1 M potassium bicarbonate, centrifuged (30 min, 3,220 × *g* and 4°C), and the supernatant used to determine tissue metabolites. Tissue lactate and amino acid levels were determined spectrophotometrically as described for plasma. Tissue glycogen concentration was assessed with amino glucosidase as described (Keppler and Decker, 1974). Glucose obtained after glycogen breakdown (after subtraction of free glucose levels) was determined with a commercial kit (Spinreact, see before). Muscle water content was analyzed by dehydrating pre-weighted muscle at 65°C until achieving constant weight (around 48 h). The percentage of water was calculated as the difference in weight between the fresh and the dry muscle divided by the fresh weight (Barragán-Méndez et al., 2018).

All assays were carried out using a PowerWave^TM^ 340 microplate spectrophotometer (Bio-Tek Instruments, Winooski, VT, United States) using KCjunior^TM^ data analysis software for Microsoft^®^.

### Statistics

Normality and homogeneity of variances were analyzed using the Shapiro–Wilk’s test and the Levene’s test, respectively. Two-way nested ANOVA test was performed with trawl (as the nested factor for each season) and time as the factors of variance. No significant differences were described due to trawls in any of the dependant variables. Thus, differences between groups were tested using one-way ANOVA with recovery time (0, 24 and 48 h or 0, 6, and 24 h) as independent variables. When necessary, data was logarithmically transformed to fulfill the requirements for ANOVA. Tukey *post hoc* test was used to identify significant differences between groups. Differences between survival rates and concentration of the physiological parameters between the groups at 0 and 24 h were evaluated by Student’s *t*-test. In addition, to establish the effect of the season, differences in plasma and muscle parameters between survivors at time 24 h after capture in spring and autumn, were also evaluated by Student’s *t*-test. Statistical significance was accepted at *p* < 0.05. All the results are represented as mean ± SEM.

## Results

### Survival Rates in Spring and Autumn

*Eledone moschata* and *O. vulgaris* did not show different survival rates between seasons despite the differences observed in the environmental variables described at the sea bottom, the aquariums and the air at the fishing deck (Table 2). *E. moschata* showed a survival rate of 100.0 ± 0.0% (91 animals captured in 13 hauls) and 92.7 ± 3.9% (83 animals captured in 10 hauls) in spring and autumn, respectively. *E. cirrhosa* was only captured in spring and its survival rate was 73.3 ± 14.1% (49 animals captured in 11 hauls). In the case of *O. vulgaris* its survival rate was 76.0 ± 11.0% in spring (72 animals captured in 12 hauls) and 75.7 ± 4.3% in autumn (44 animals captured in 7 hauls; mean ± SEM). All mortality occurred within the first 8 h after capture. We were unable to spot any major difference (external injuries or physical damage) between alive or dead animals.

**TABLE 2:** Survival rates of *E. moschata*, *E. cirrhosa*, and *O. vulgaris* captured during bottom-trawling in the Gulf of Cadiz (Spain).

**Species**	**Season**	**Survival (%)**	**Trawls (*n*)**	**Animals (*n*)**
*E. moschata*	Spring	100.0 ± 0.0	13	91
	Autumn	92.7 ± 3.9	10	83
*E. cirrhosa*	Spring	73.3 ± 14.1	11	49
*O. vulgaris*	Spring	76.0 ± 11.0	12	72
	Autumn	75.7 ± 4.3	7	44

*The table shows the number of trawls included and the total number of animals employed in every experiment. Data are represented as mean ± SEM. No statistical differences were described in the survival rates between spring and autumn for any species (Student’s *t*-test, *p* < 0.05).*

### Physiological Recovery Curve

**FIGURE 2:**
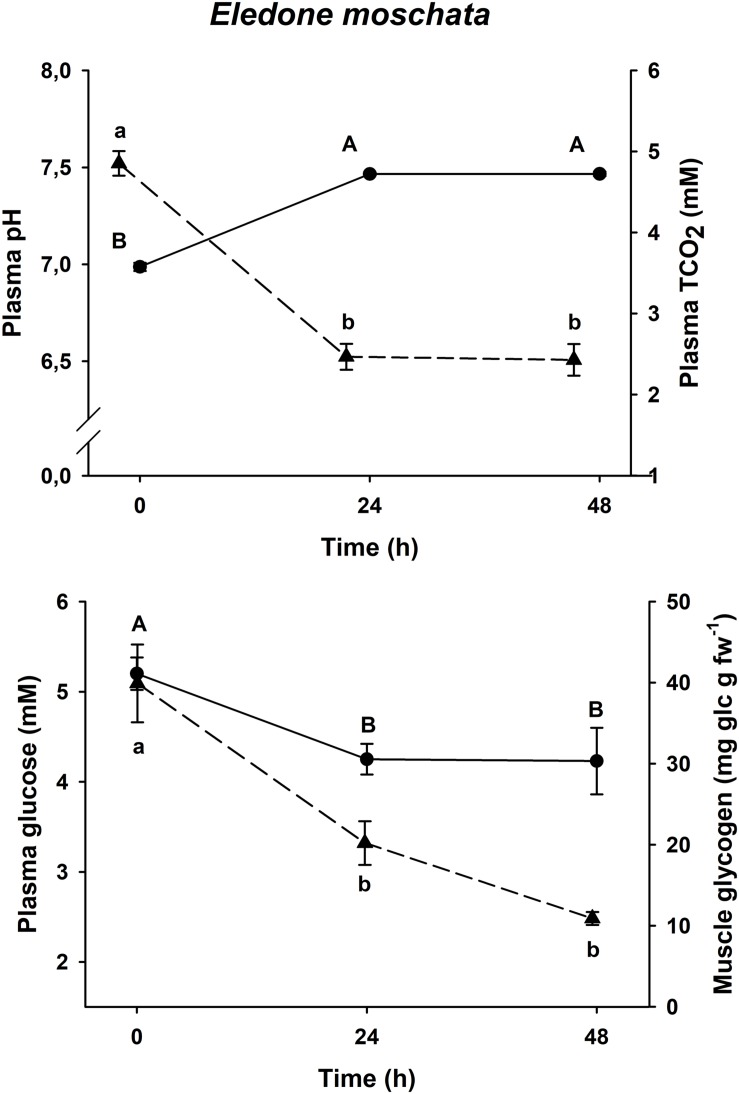
Time course recovery of plasma pH (continuous line) and TCO_2_ (dashed line) (upper figure) and plasma glucose (continuous line) and muscle glycogen (dashed line) (lower figure) in bottom trawled *E. moschata* in the Gulf of Cadiz (Spain). Plasma and muscle samples were taken just after bottom trawling (0 h) and at different recovery times (24 and 48 h). Data are expressed as mean ± SEM (*n* = 18–19 per group). Different capital letters indicate significant differences between groups for plasma pH and glucose, while different lowercase letters indicate significant differences between groups for plasma TCO_2_ and muscle glycogen (one-way ANOVA followed by a *post hoc* Tukey test, *p* < 0.05). *fw* means fresh weight.

**FIGURE 3:**
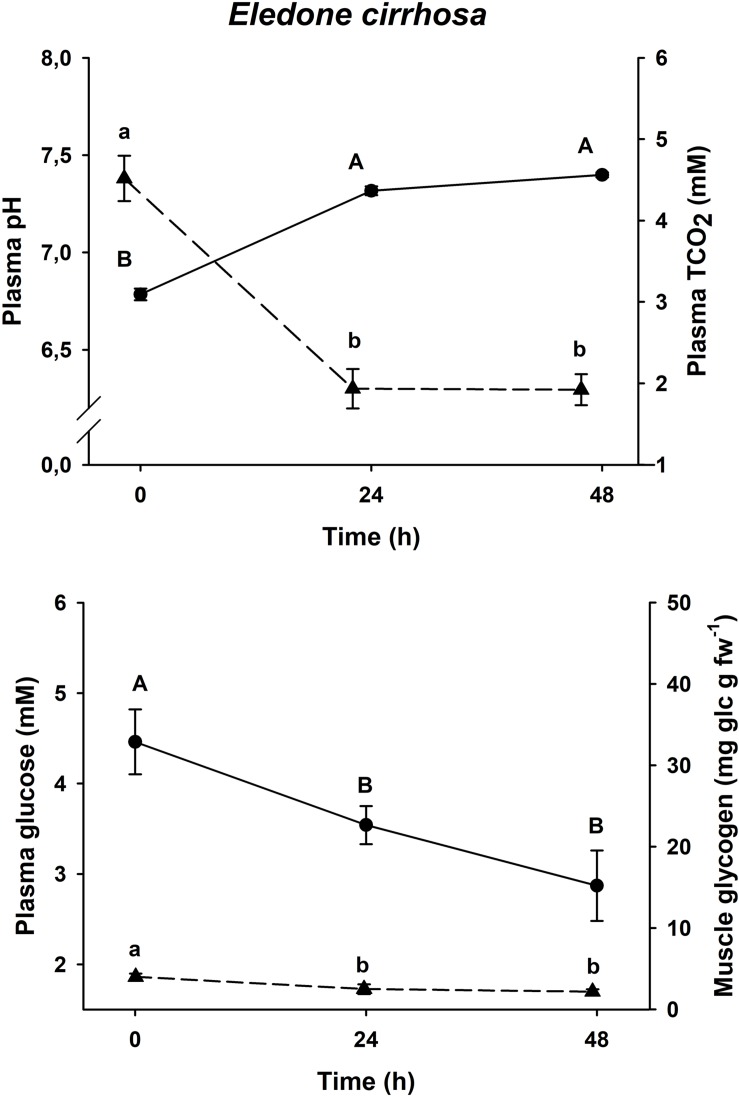
Time course recovery of plasma pH (continuous line) and TCO2 (dashed line) (upper figure) and plasma glucose (continuous line) and muscle glycogen (dashed line) (lower figure) in bottom trawled *E. cirrhosa* in the Gulf of Cadiz (Spain). Plasma and muscle samples were taken just after bottom trawling (0 h) and at different recovery times (24 and 48 h). Data are expressed as mean ± SEM (*n* = 12 per group). Different capital or lowercase letters indicate significant differences between groups for the same variable (one-way ANOVA followed by a *post hoc* Tukey test, *p* < 0.05). More details are described in Figure 2.

**FIGURE 4:**
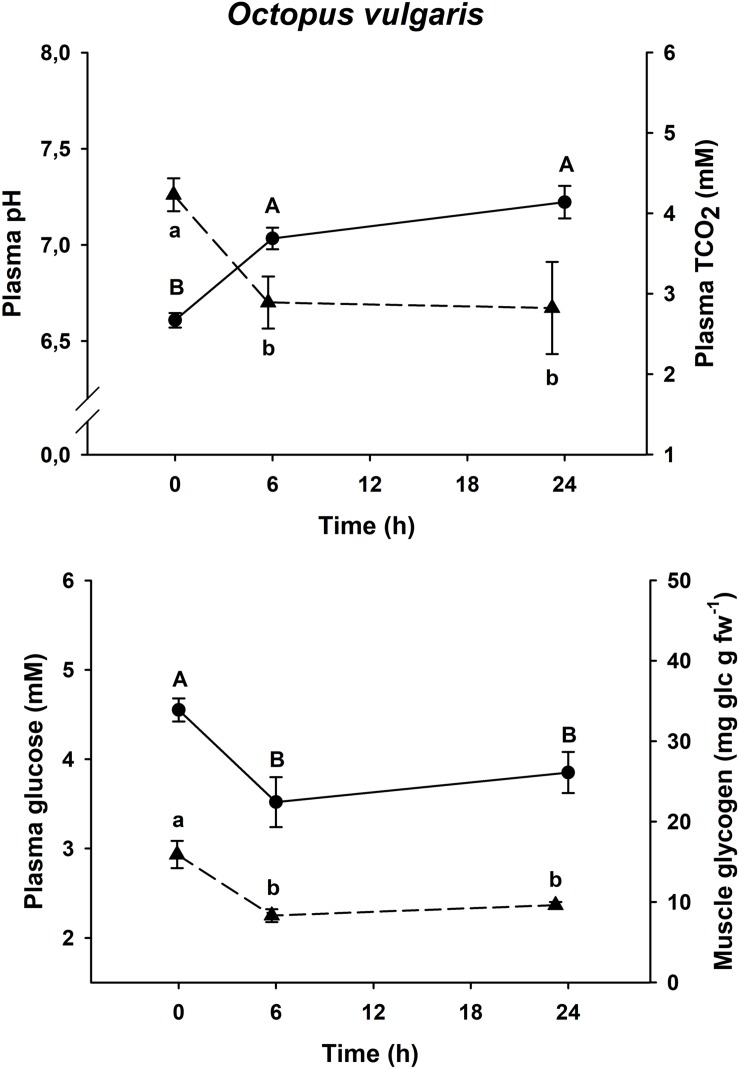
Time course recovery of plasma pH (continuous line) and TCO2 (dashed line) and plasma glucose (continuous line) and muscle glycogen (dashed line) (upper figure) and (lower figure) in bottom trawled *O. vulgaris* in the Gulf of Cadiz (Spain). Plasma and muscle samples were taken just after bottom trawling (0 h) and at different recovery times (6 and 24 h). Data are expressed as mean ± SEM (*n* = 14–15 per group). Different capital or lowercase letters indicate significant differences between groups for the same variable (one-way ANOVA followed by a *post hoc* Tukey test, *p* < 0.05). More details are described in Figure 2.

Physiological changes in plasma glucose, muscle glycogen and plasma pH and TCO_2_ after an acute-stress situation, and further recovery in onboard tanks in *E. moschata*, *E. cirrhosa*, and *O. vulgaris* are shown in Figures 2–4, respectively.

All three species shown the same responses for all four variables. Plasma pH and TCO_2_ changes were shared by all three species, with minimum pH and maximum TCO_2_ at time 0 h (*p* < 0.05), and maximum pH and minimum TCO_2_ at all other times (*p* < 0.05, one-way ANOVA followed by a Tukey *post hoc* test). Maximum levels of plasma glucose and muscle glycogen are described at time 0 h, immediately before being introduced into the recovery aquariums. Both *Eledone* spp. showed statistically lower concentrations at times 24 h and 48 h after recovery (without differences between both groups, *p* < 0.05, one-way ANOVA followed by a Tukey *post hoc* test), in the same way as *O. vulgaris* showed statistically lower values at times 6 and 24 h after recovery (without differences between both groups, *p* < 0.05).

Other physiological parameters analyzed in plasma and muscle in a time-course following recovery in onboard aquariums after bottom-trawling are shown in Table 3 (*E. moschata*), Table 4 (*E. cirrhosa*), and Table 5 (*O. vulgaris*).

**TABLE 3:** Physiological parameters in plasma and muscle of *E. moschata* after bottom trawling in spring 2017.

**Parameter**	**Time post-recovery**		
	**0 h**	**24 h**	**48 h**
Plasma lactate (mM)	0.35 ± 0.04	0.36 ± 0.02	0.35 ± 0.04
Plasma amino acids (mM)	21.8 ± 1.0 A	19.6 ± 0.9 AB	16.0 ± 1.7 B
Plasma hemocyanin (mM)	0.94 ± 0.05 B	1.56 ± 0.06 A	1.48 ± 0.08 A
Plasma lysozyme (μg mL^–1^)	0.38 ± 0.05 A	0.19 ± 0.03 B	0.20 ± 0.04 B
Muscle glucose (mg glc g fw^–1^)	23.3 ± 2.5	27.4 ± 2.2	24.1 ± 2.2
Muscle lactate (μg g fw^–1^)	31.3 ± 3.5	26.9 ± 2.9	29.6 ± 2.8
Muscle amino acids (μmol g fw^–1^)	202 ± 23 A	158 ± 9 AB	134 ± 12 B
Muscle water (%)	78.7 ± 0.2	78.7 ± 0.3	79.4 ± 0.5
			

*Samplings were done during a time course after recovery in onboard tanks (0, 24 and 48 h). Data is shown as mean ± SEM (*n* = 18–19 per group). Differences between sampling times are indicated with different capital letters (*p* < 0.05, one-way ANOVA followed by a Tukey’s *post hoc* test). *fw* means fresh weight.*

**TABLE 4:** Physiological parameters in plasma and muscle of *E. cirrhosa* after bottom trawling in spring 2017.

**Parameter**	**Time post-recovery**		
	**0 h**	**24 h**	**48 h**
Plasma lactate (mM)	0.45 ± 0.07	0.56 ± 0.06	0.48 ± 0.07
Plasma amino acids (mM)	18.4 ± 2.0 A	12.3 ± 1.7 B	11.9 ± 1.2 B
Plasma hemocyanin (mM)	0.81 ± 0.03	0.95 ± 0.07	0.98 ± 0.11
Plasma lysozyme (μg mL^–1^)	0.35 ± 0.03	0.35 ± 0.04	0.37 ± 0.04
Muscle glucose (mg glc g fw^–1^)	11.5 ± 1.6 A	7.7 ± 1.7 AB	5.2 ± 2.0 B
Muscle lactate (μg g fw^–1^)	13.0 ± 3.2	14.5 ± 4.3	14.6 ± 2.6
Muscle amino acids (μmol g fw^–1^)	123 ± 9	128 ± 7	129 ± 6
Muscle water (%)	78.1 ± 0.3 B	78.2 ± 0.2 AB	79.9 ± 1.2 A
			

*Samplings were done during a time course after recovery in onboard tanks (0, 24, and 48 h). Data is shown as mean ± SEM (*n* = 12 per group). Differences between sampling times are indicated with different capital letters (*p* < 0.05, one-way ANOVA followed by a Tukey’s *post hoc* test). *fw* means fresh weight.*

**TABLE 5:** Physiological parameters in plasma and muscle of *O. vulgaris* after bottom trawling in autumn 2017.

**Parameter**	**Time post-recovery**		
	**0 h**	**6 h**	**24 h**
Plasma lactate (mM)	0.22 ± 0.01	0.24 ± 0.03	0.28 ± 0.06
Plasma amino acids (mM)	16.7 ± 0.9 A	13.7 ± 2.9 AB	8.2 ± 1.8 B
Plasma hemocyanin (mM)	0.87 ± 0.01 B	0.83 ± 0.04 B	1.18 ± 0.06 A
Plasma lysozyme (μg mL^–1^)	0.17 ± 0.02 A	0.11 ± 0.02 B	0.05 ± 0.02 B
Muscle glucose (mg glc g fw^–1^)	26.3 ± 2.7	18.5 ± 3.9	26.9 ± 5.3
Muscle lactate (μg g fw^–1^)	24.1 ± 3.7	25.9 ± 3.1	18.7 ± 4.9
Muscle amino acids (μmol g fw^–1^)	81.8 ± 6.9	95.9 ± 7.2	99.6 ± 8.3
Muscle water (%)	79.8 ± 0.3	78.8 ± 0.6	79.2 ± 0.2
			

*Samplings were done during a time course after recovery in onboard tanks (0, 6, and 24 h). Data is shown as mean ± SEM (*n* = 14–15 per group). Differences between sampling times are indicated with different capital letters (*p* < 0.05, one-way ANOVA followed by a Tukey’s *post hoc* test). *fw* means fresh weight.*

Plasma and muscle lactate did not show statistical changes in any of the species tested (*p* < 0.05). Plasma amino acids show maximum concentration at time 0 h in all three species, and minimum concentrations at the end of the experiment, without statistical differences between the last two sampling times in all species (*p* < 0.05). Plasma Hc shows minimum concentrations at time 0 h in *E. moschata* and *O. vulgaris*, and maximum concentrations in all other times, without significant differences between the last two recovery times (*p* < 0.05). Hc in *E. cirrhosa* did not change along the experiment. After 24 h recovery, Hc values were 1.56 ± 0.06 mM in *E. moschata*, 0.95 ± 0.07 mM in *E. cirrhosa*, and 1.18 ± 0.06 mM in *O. vulgaris*. Plasma lysozyme activity shows the opposite profile than plasma Hc, with maximum concentrations at time 0 h in *E. moschata* and *O. vulgaris*, and minimum concentrations in all other times, without significant differences between the last two recovery times (*p* < 0.05). Lysozyme activity in *E. cirrhosa* did not change along the experiment.

Muscle glucose only showed statistical changes in *E. cirrhosa*, with maximum concentrations at time 0 h, and minimum at time 48 h (*p* < 0.05). Muscle amino acids only showed statistical changes in *E. moschata*, with maximum concentrations at time 0 h, and minimum at time 48 h (*p* < 0.05). Muscle water content only showed statistical changes in *E. cirrhosa*, with its minimum percentage at time 0 h, and maximum at time 48 h (*p* < 0.05). After 24 h recovery, muscle water content was 78.7 ± 0.3% in *E. moschata*, 78.2 ± 0.2% in *E. cirrhosa*, and 79.2 ± 0.2% in *O. vulgaris*.

### Physiological Differences Between Spring and Autumn

*Eledone moschata* was sampled in spring and autumn and physiological recovery was analyzed by sampling plasma and muscle at times 0 and 24 h after recovery in onboard aquariums (Table 6); *E. cirrhosa* was only sampled in spring at times 0 and 24 h (Table 7); and *O. vulgaris* was sampled in spring (Table 8); and autumn (Figure 4 and Table 5).

**TABLE 6:** Plasma and muscle physiological parameters in *E. moschata* immediately after bottom trawling in the Gulf of Cadiz (0 h) and 24 h after recovery in water tanks in spring 2018 and autumn 2017.

**Parameter**	**Spring**		**Autumn**	
	**0 h**	**24 h**	**0 h**	**24 h**
Plasma glucose (mM)	5.6 ± 0.1	4.5 ± 0.2^*^	5.4 ± 0.6	3.8 ± 1.6^*#^
Plasma lactate (mM)	0.37 ± 0.03	0.38 ± 0.03	0.68 ± 0.26^#^	0.60 ± 0.24^#^
Plasma amino acids (μmol g fw^–1^)	15.8 ± 1.0	12.5 ± 1.0^*^	13.9 ± 0.7^#^	11.7 ± 0.7^*^
Plasma proteins (mg mL^–1^)	75.6 ± 2.8	75.2 ± 2.6	N/A	N/A
Plasma hemocyanin (mM)	0.84 ± 0.04	1.06 ± 0.06^*^	0.86 ± 0.01	1.34 ± 0.05^*#^
Plasma lysozyme (μg mL^–1^)	0.11 ± 0.02	0.04 ± 0.00^*^	0.15 ± 0.11	0.14 ± 0.09^#^
Plasma peroxidase (U mL^–1^)	6.5 ± 0.6	5.3 ± 0.5	N/A	N/A
Plasma protease (%)	0.86 ± 0.42	0.0 ± 0.0^*^	N/A	N/A
Plasma antiprotease (%)	0.94 ± 0.11	3.52 ± 0.81^*^	N/A	N/A
Plasma total PO-like activity (mU mL^–1^)	41.9 ± 3.0	57.3 ± 5.0^*^	N/A	N/A
Muscle glucose (mg glc g fw^–1^)	44.8 ± 3.6	47.3 ± 5.2	49.6 ± 3.3	56.7 ± 4.2
Muscle glycogen (mg glc g fw^–1^)	45.8 ± 6.4	22.0 ± 5.0^*^	60.9 ± 5.4	40.4 ± 3.5^*#^
Muscle lactate (μg g fw^–1^)	14.7 ± 3.7	9.9 ± 3.1	38.6 ± 1.9^#^	39.7 ± 2.2^#^
Muscle amino acids (μmol g fw^–1^)	67.9 ± 8.0	44.3 ± 6.6^*^	75.4 ± 2.4^#^	74.8 ± 3.1^#^
Muscle water (%)	84.1 ± 0.6	82.5 ± 0.5	78.9 ± 1.3	80.1 ± 1.5^*#^

Data is shown as mean ± SEM (*n* = 43 and 41 per group in spring and autumn, respectively). Asterisks (^*^) indicate significant differences between 0 h and 24 h for each season (*p* < 0.05, Student’s *t*-test). Hashtags (#) indicate significant differences between spring and autumn for groups sampled at the same time (*p* < 0.05, Student’s *t*-test). N/A, not available. *fw* means fresh weight.

**TABLE 7:** Plasma and muscle physiological parameters in *E. cirrhosa* immediately after bottom trawling in the Gulf of Cadiz (0 h) and 24 h after recovery in water tanks in spring 2018.

**Parameter**	**Time post-recovery**	
	**0 h**	**24 h**
Plasma glucose (mM)	3.9 ± 0.1	2.7 ± 0.2^*^
Plasma lactate (mM)	0.39 ± 0.03	0.32 ± 0.03
Plasma amino acids (μmol g fw^–1^)	42.2 ± 2.6	24.8 ± 2.7^*^
Plasma proteins (mg mL^–1^)	82.7 ± 1.7	56.7 ± 3.9^*^
Plasma hemocyanin (mM)	0.80 ± 0.06	0.74 ± 0.09
Plasma lysozyme (μg mL^–1^)	0.11 ± 0.03	0.10 ± 0.02
Plasma peroxidase (U mL^–1^)	9.4 ± 1.0	4.2 ± 0.5^*^
Plasma protease (%)	12.3 ± 2.4	0.0 ± 0.0^*^
Plasma antiprotease (%)	13.8 ± 0.16	2.42 ± 0.44^*^
Plasma total PO-like activity (mU mL^–1^)	53.7 ± 6.4	76.2 ± 11.2
Muscle glucose (mg glc g fw^–1^)	21.0 ± 2.2	14.9 ± 1.6^*^
Muscle glycogen (mg glc g fw^–1^)	3.5 ± 0.4	1.9 ± 0.4^*^
Muscle lactate (μg g fw^–1^)	10.6 ± 4.7	7.3 ± 5.0
Muscle amino acids (μmol g fw^1^)	123 ± 6	110 ± 6
Muscle water (%)	85.9 ± 1.0	83.0 ± 0.6^*^

Data is shown as mean ± SEM (*n* = 20–29 per group). Asterisks (^*^) indicate significant differences between 0 h and 24 h (*p* < 0.05, Student’s *t*-test). *fw* means fresh weight.

**TABLE 8:** Plasma and muscle physiological parameters in *O. vulgaris* immediately after bottom trawling in the Gulf of Cadiz (0 h) and 24 h after recovery in water tanks in spring 2018.

**Parameter**	**Time post-recovery**	
	**0 h**	**24 h**
Plasma glucose (mM)	5.4 ± 0.2^#^	3.6 ± 0.2^*^
Plasma lactate (mM)	0.27 ± 0.02	0.22 ± 0.01
Plasma amino acids (μmol g fw^–1^)	19.0 ± 2.0	13.4 ± 1.5^*^
Plasma proteins (mg mL^–1^)	92.9 ± 1.5	75.6 ± 5.5^*^
Plasma hemocyanin (mM)	0.80 ± 0.02	1.13 ± 0.08^*^
Plasma lysozyme (μg mL^–1^)	0.16 ± 0.02^#^	0.07 ± 0.01^*^
Plasma peroxidase (U mL^–1^)	5.4 ± 0.7	3.2 ± 0.3^*^
Plasma protease (%)	43.3 ± 2.9	22.2 ± 2.2^*^
Plasma antiprotease (%)	0.46 ± 0.04	0.66 ± 0.06^*^
Plasma total PO-like activity (mU mL^–1^)	12.2 ± 1.5	20.5 ± 3.4^*^
Muscle glucose (mg glc g fw^–1^)	34.0 ± 2.1	18.2 ± 2.7^*^
Muscle glycogen (mg glc g fw^–1^)	5.4 ± 0.7^#^	2.0 ± 0.3^*#^
Muscle lactate (μg g fw^–1^)	15.7 ± 3.5	8.8 ± 4.2
Muscle amino acids (μmol g fw^–1^)	115 ± 7^#^	95 ± 6 ^*^
Muscle water (%)	81.5 ± 0.4^#^	83.0 ± 0.6^#^

Data is shown as mean ± SEM (*n* = 25–36 per group). Asterisks (^*^) indicate significant differences between 0 h and 24 h (*p* < 0.05, Student’s *t*-test). Hashtags (#) indicate significant differences between spring and autumn for groups sampled at the same time (*p* < 0.05, Student’s *t*-test). *fw* means fresh weight.

#### *E. moschata* (Table 6)

Plasma glucose was significantly lower at time 24 h in autumn when compared to spring (3.8 ± 1.6 mM vs. 4.5 ± 0.2 mM). Plasma lactate in autumn at both sampling times shown almost double concentration than in spring (above 0.60 mM in autumn, and circa 0.37–0.38 mM in spring). Plasma amino acids only show differences due to season at time 0 h, with lower concentration in autumn. Plasma proteins shown no changes due to sampling time in spring. Plasma hemocyanin and lysozyme activity displayed significant differences due to season only at time 24 h, with higher values in autumn than in spring. Other immune system parameters in plasma were only analyzed in spring and will be employed to compare inter-specific differences between all three octopus spp. No changes are also described for muscle glucose with either time or season. However, muscle glycogen shown significantly higher values at time 24 h in autumn that in spring (40.4 ± 3.5 and 22.0 ± 5.0 mg glc g^–1^ fw, respectively). Muscle lactate and amino acids were significantly higher at both sampling times in autumn than in spring. Water content in the muscle show differences due to season only at time 24 h, with 80.1 ± 1.5% in autumn, and 82.5 ± 0.6% in spring.

#### *E. cirrhosa* (Table 7)

As *E. cirrhosa* was only sampled in spring, no comparison was possible between spring and autumn. The analyzed physiological parameters from samples collected in spring 2017 and 2018 are similar. Plasma glucose, amino acids, proteins and peroxidase activity, as well as muscle glucose, glycogen and water content shown higher values at time 0 h than at time 24 h. No changes are observed due to sampling time in plasma lactate, hemocyanin lysozyme and muscle lactate and amino acids (*p* < 0.05, Student’s *t*-test).

#### *O. vulgaris* (Table 8)

Plasma glucose at time 0 h in spring was significantly higher than in autumn (5.4 ± 0.2 mM and 4.5 ± 0.1mM, respectively), and no differences are described at time 24 h between seasons. Plasma lactate shown changes neither with sampling time nor due to season. Plasma amino acids shown no statistical differences due to season but to sampling time, with higher concentrations at time 0 h. Plasma proteins, analyzed only in spring, shown higher values at time 0 h. Plasma hemocyanin was statistically similar between season for each sampling time. Plasma lysozyme show higher activity rate in autumn than in spring at time 0 h (0.17 ± 0.02 and 0.16 ± 0.02 μg mL^–1^, respectively). Plasma peroxidase was only analyzed in spring, and its activity was higher at time 0 h than at time 24 h (5.4 ± 0.7 and 3.2 ± 0.3 U mL^–1^, respectively). Muscle glucose did not show differences between seasons at any sampling time. Glycogen in muscle showed statistically differences at time 0 h between spring and autumn (5.4 ± 0.7 and 15.9 ± 1.7 mg glc g^–1^ wet weight, respectively) and at time 24 h (2.0 ± 0.3 and 9.6 ± 0.4 mg glc g^–1^ wet weight, respectively). Lactate levels did not present differences between time or season. In the case of amino acids in muscle a significant decrease was observed 24 h after recovery in spring (115 ± 7 μmol g^–1^ wet weight after fishing and 95 ± 6 μmol g^–1^ wet weight after 24 h). Differences at time 0 h between seasons were also showed, as muscle amino acids in autumn were 81.8 ± 6.9 μmol g^–1^ wet weight. Finally, muscle water content show differences only due to season, with values at time 0 h of 81.5 ± 0.4% in spring and 79.8 ± 0.3% in autumn; and values at time 24 h of 83.0 ± 0.6% in spring and 79.2 ± 0.2% in autumn.

### Changes in the Color of Haemolymph

**FIGURE 5:**
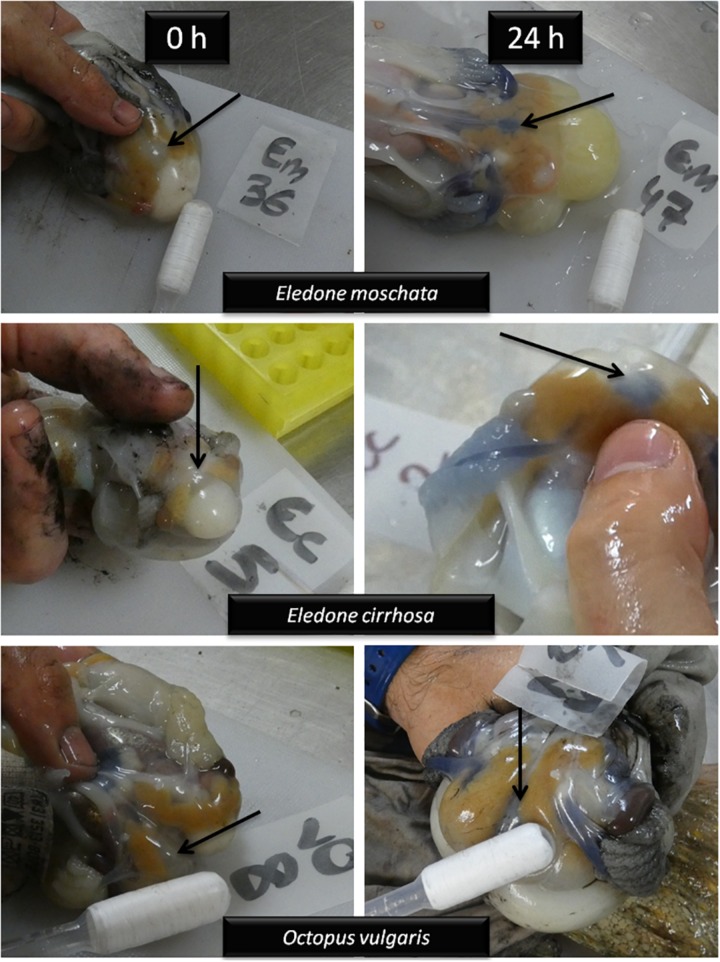
Detailed photographs of the circulatory system of *E. moschata*, *E. cirrhosa*, and *O. vulgaris* captured by bottom trawling at times 0 and 24 h after recovery into onboard tanks. Immediately after capture (0 h) haemolymph is translucent, and turned into turquoise blue 24 h after recovery, evidencing reduced and oxidized hemocyanin, respectively. Arrows indicate ventral heart.

By taking pictures of sampled octopus, framing the ventral heart and the circulatory system, we have been able to clearly distinguish two hemocyanin states: oxidized and reduced (Figure 5). Those animals at time 0 h presented a translucent haemolymph, which correspond to a reduced hemocyanin under circulatory conditions. Moreover, in those animals that survived for 6 h or more, the haemolymph acquires a very striking turquoise blue color, as indicative of an oxidized state of the hemocyanin.

### Immune System Differences in Plasma: Comparison Between Species

Several plasma parameters were analyzed in all three species at times 0 and 24 h in spring (Tables 6–8).

Plasma hemocyanin and total PO-like activity displayed circa 33% lower values at time 0 h in *E. moschata* and *O. vulgaris*. Lysozyme activity showed changes due to sampling time only in *E. moschata* and *O. vulgaris*, with higher values at time 0 h in both species. Statistically higher peroxidase activity and plasma concentration of proteins were described in *E. cirrhosa* and *O. vulgaris* but in *E. moschata* at time 0 h. Proteases showed higher activities in all three species at time 0 h, reaching values of 0.86 ± 0.42, 12.3 ± 2.4, and 43.3 ± 2.9% in *E. moschata*, *E. cirrhosa*, and *O. vulgaris*, respectively; and values at time 24 h of 0.0 ± 0.0% in both *Eledone* spp., and 22.2 ± 2.2% in *O. vulgaris*. Antiproteases (antitrypsin activity) in all three species, otherwise, showed statistically higher activity at time 24 h, with values around 0.46 to 1.38 mU mL^–1^ at time 0 h, and 2–4 mU mL^–1^ in both *Eledone* spp., and 0.66 ± 0.06 mU mL^–1^ in *O. vulgaris*.

## Discussion

The present pilot study serves to unravel physiological consequences of an acute-stress situation in Octopodidae species. Taking advantage of animals captured by trawling, which is considered a source of acute-stress in marine organisms (Lund et al., 2009; Gallagher et al., 2014; Veldhuizen et al., 2018), we have managed to decipher some of these mechanisms through the physiological stability of octopus. Survival rates of three species (*E. moschata*, *E. cirrhosa*, and *O. vulgaris*) captured in spring and autumn in South Western Atlantic waters of Europe were evaluated, and physiological responses related to energy management and the immune system were assessed. This study described that octopus rely on amino acids and carbohydrates to face an acute-stress challenge, and that the immune system is compromised during the first hours after capture, but managed to recover in less than 24 h. However, to the best of our knowledge, this study is the first to describe, in juvenile and adult octopus, haemolymph and muscle physiological parameters after a dramatic event such as being trawled and exposed to air. These results can be useful to improve aquaculture conditions through the recognition of stress situations that may affect welfare.

### Survival Rates

Survival rates were evaluated in three Octopodidae species in spring and autumn after 1 h of being trawled followed by 65 min of air exposure (mimicking commercial procedures). After such a dramatic event, octopus were allowed to recover in onboard aquariums, showing differentiated survival rates 24 h later depending on the species: *E. moschata* (as high as 100% survival in spring, and 92% in autumn), *E. cirrhosa* (circa 73% in spring) and *O. vulgaris* (above 75% in both spring and autumn). In light of these results, there is a lack of differences in the survival rates for each species between spring and autumn. However, it has been described before that invertebrates such as crustaceans and bivalves presented higher mortality rates in months with higher water temperatures (Raicevich et al., 2014; Mehault et al., 2016; Clements et al., 2018). The fact that octopuses had no differences between survival rates due to environmental temperature changes (within the range evaluated in this study, and 1.0–4.5°C difference between the bottom of the ocean and the aquariums) could indicate a high capacity to acclimate to different environmental conditions, as suggested before for *O. vulgaris* embryo and paralarvae (Garrido et al., 2017; Nande et al., 2018).

The novelty of this study relies on the evaluation of the survival rates in discarded octopus under fisheries conditions. Previously, only one study evaluated survival rates of captured octopus (Conners and Levine, 2017). These authors studied the giant Pacific octopus (*Enteroctopus dofleini*) captured by pot-fishing and trawling, and evaluated its condition factor after capture. However, following their criteria for assessing condition of octopus immediately after capture, most of the animals collected in the present study should be considered dead (though survival rates 24 h later were above 73% in all three species), as they did not move in response to handling, they show color fading to gray or white, and show flaccid arms and suckers not adhering to surfaces (personal observations done during the experiments described herein). Thus, we considered necessary to conduct experiments where captured octopus are allowed to recover in appropriate water conditions for a few hours to evaluate survival rates. We hypothesize that bottom trawling under the circumstances described in the present study was such an intense process (lasting almost 2 h, including trawling and air exposure) that animals were exhausted when observed for the first time (prior to sampling and/or introducing into recovery aquariums). Moreover, Conners and Levine (2017) maintained a few animals in onboard tanks and observed no delayed mortality rates after 24 h recovery. In our study, all deceased octopus were considered dead in less than 8 h after being introduced into the aquariums. Coinciding with other taxa, survivors (after an acute-stress situation such as being captured and exposed to air) seem to be recovered in a few hours (Barragán-Méndez et al., unpublished) (Bergmann et al., 2001; Costas et al., 2011; Skrzynska et al., 2018; Barragán-Méndez et al., 2019). In this sense, the analysis of certain physiological parameters seems to be a good proxy to evaluate recovery. This study aims at evaluating the maximum survival rates of these species after capture (“maximum” as the animals were recovered in individual tanks with environmental conditions similar to those in the wild). We assumed that immediately discarded octopus would fall prey to predation rapidly after being returned to the ocean due to their catatonic state after capture. However, future advances should be focused on the way of diminishing dead of discards due to predators as, for example, releasing the animals through a tube submerged a few meters below the ocean surface to reduce bird’s capture, or allowing captured octopus to be recovered in onboard tanks for a certain period of time before being discarded.

### pH, TCO_2_, and Hypoxia

In this study, blood pH and TCO_2_ were altered by bottom-trawling and further recovery in onboard tanks, suggesting a metabolic acidosis after an acute-stress episode altogether with an anaerobic situation, as described before for other aquatic animals (Mandic and Regan, 2018). It was stated that maintaining constant levels of these parameters in intra- and extracellular fluids of marine organisms are of paramount importance for the correct maintenance of the acid-base balance (Tresguerres and Hamilton, 2017). Thus, in this study, survivors of all three Octopodidae species managed to presumably recover plasma basal pH and TCO_2_ levels in less than 24 h after an acute-stress situation as seen by the lack of differences between the last sampling points in the described experiments.

It should be mention that pH of the haemolymph and environmental temperature contribute to the oxygen affinity of dissolved Hc (Oellermann et al., 2015a, b). It was stated that *E. moschata* shows 100% oxygen binding to Hc (at temperatures similar to those described in this study) at plasma pH 7.5, while the maximal pH-dependent release of oxygen by Hc occurs at pH 7.0 (Oellermann et al., 2015a). Thus, our results of plasma pH confirmed that photographs taken of *Eledone* spp. and *O. vulgaris* in this study after an acute-stress challenge evidenced a deep blood anaerobiosis, as translucent haemolymph could be thus associated to 0% oxygen being transported by Hc, while octopus maintained in water aquariums for more than 6 h show maximum oxygen linked to Hc as seen by the deep-turquoise color of haemolymph. Our results show lower plasma pH levels in *O. vulgaris* compared to *Eledone* spp. This fact may define tolerance to hypoxia in these species, and also be a reason for the higher mortality rates of *O. vulgaris* when compared to *E. moschata* captured in the same geographical area (similar depths and water temperatures). However, further studies should be conducted to locate the causes of death of these species after a stress like the one described here.

Another striking result is that plasma TCO_2_, mostly in the form of HCO_3_^–^ at circulating pH conditions in the blood (Boutilier et al., 1985) in all three Octopodidae species, is as low as 2–3 mM at pH 7.4–7.5; while marine crustaceans show plasma HCO_3_^–^ levels around 10–20 mM (at plasma pH 7.5–7.8 and water temperature 15–20°C) (Howell et al., 1973); teleost fish show 5–10 mM HCO_3_^–^ in plasma at a pH around 7.6–7.8 (Boutilier et al., 1985); and mammals evidenced higher plasma HCO_3_^–^ concentrations around 20 mM at pH 7.4 (Pamenter et al., 2018). As maintenance of blood pH in vertebrates is mostly controlled by plasma bicarbonate (Tresguerres and Hamilton, 2017), plasma of octopus may be buffered by proteins such as Hc, which constitutes 98% of all proteins in plasma of these species (Aguila et al., 2007). This low bicarbonate levels in cephalopods may be of interest to further investigate the formation of their hard structures and thus evaluate growth rates (Arkhipkin et al., 2018), and/or to describe their acid-base mechanisms.

### Immune System

The immune system of all three studied Octopodidae was also altered due to an acute-stress situation, with species-specific differences. It was stated that the general health status of octopus is related to the activation of immune mechanisms facing harmful situations (Roumbedakis et al., 2017). Thus, lower Hc concentrations in plasma of *E. moschata* and *O. vulgaris* in this study may be associated to the conversion of Hc into a phenoloxidase (PO)-like enzyme, as was described for crustaceans and other invertebrates (Adachi et al., 2003; Decker and Jaenicke, 2004). Supporting this idea, some studies conducted in cuttlefish (*Sepia officinalis*) embryo shown increased PO activity after exposure to dissolved metals (Lacoue-Labarthe et al., 2009), while *O. vulgaris* infected by a protozoan parasite show changes in blood Hc (Castellanos-Martinez et al., 2014). In the present study, plasma Hc reached stable values circa 1–1.5 mM in all animals after 24 h recovery, similar to those levels described in several cephalopod species (Senozan et al., 1988; Aguila et al., 2007; Sancho et al., 2015; Roumbedakis et al., 2017). Our hypothesis that Hc acts as a total PO-like enzyme in octopus is based in the fact that after bottom trawling *E. moschata* and *O. vulgaris* had 60–80% less Hc and pro-PO activity than 24 h later, when recovered. Future studies, based in these results and actual knowledge in arthropods, may be considered to explore the Hc involvement in the immune system in cephalopods.

Plasma lysozyme in *E. moschata* and *O. vulgaris* showed higher activities immediately after the acute-stress situation, maintaining lower and stable values after 24 and 6 h, respectively. Lysozyme is an essential component of the innate immunity of octopus and part of their antibacterial mechanism (Malham et al., 1998b), and it was described not only in plasma but also in other tissues (Malham et al., 1998b; Martinez-Montaño et al., 2017). As for Hc, lysozyme activity shows no changes in *E. cirrhosa*. The reason for this lack of lysozyme response was unknown, though the activity of this enzyme in both *Eledone* spp. is similar. However, it was described in *E. cirrhosa* that injection of *Vibrio anguillarum* caused an increase in lysozyme activity in the branchial heart without changes in the activity of this enzyme in the haemolymph, while also reduced plasma antiprotease activity (Malham et al., 1998b), coinciding with our results in all three species. *E. cirrhosa* has a well develop immune system in the haemolymph, with bacteriostatic, lysozyme, protease, antiprotease and peroxidase activities, as seen by our results and as described by other authors (Malham and Runham, 1998). It was also described for this species that repeated sampling of haemolymph induced an increase in the haemocytes (Malham et al., 1998a). Thus, *E. cirrhosa* seemed to present a differentiated immune response compared to *E. moschata* and *O. vulgaris*, which may explain observed differences in the mortality rates between both *Eledone* spp. However, protease and antiprotease activities seemed to behave similarly in all three species, and may highlight shared immune responses in Octopodidae. According to these results, it can be speculated that antiproteases increased in response to the high protease activity observed at time 0 h, as an endogenous mechanism to protect host cellular damage. Interestingly, only *E. cirrhosa* specimens showed the opposite pattern, with lower antiprotease activity at 24 h, a fact that could be related to their lower survival rates. Such remarkable similarities and differences deserve future approaches to unravel the mechanisms of action in these interesting species.

Another relevant immune mechanism is the presence of peroxidase activity in plasma of the haemolymph. Peroxidase enzymes may act on the same substrates than PO (Lacoue-Labarthe et al., 2009). In this study, peroxidase activity reached similar values in plasma of all three species at time 24 h post-recovery. However, immediately after capture there is a substantial increase in this defense mechanism in *E. cirrhosa* and *O. vulgaris*. Due to the toxicity of peroxide (H_2_O_2_) molecules (substrates in the peroxidase reaction) and its precursor, the anion superoxide (O_2_^–^), these two species may face a greater oxidative stress than *E. moschata*, causing cellular injuries via protein and DNA oxidation and lipid peroxidation (Kowaltowski et al., 2009) and higher mortality rates, as described in this study. Moreover, those species evaluated in this study with the highest mortality rates (*E. cirrhosa* and *O. vulgaris*) also displayed changes in muscle water content, which is related to dehydration/overhydration processes after an acute-stress situation such as air-exposure (Barragán-Méndez et al., 2018), while *E. moschata* (with survival rates above 92%) shows no differences in muscle moisture depending on sampling time. Thus, similar stressful situations may induce differentiated responses depending on the species, and evidenced that aquaculture of octopus may require further studies focused on their physiological stress responses.

### Carbohydrates Metabolism

Muscle of invertebrates acts as a reserve for carbohydrates, stored in the form of glycogen (Lee et al., 2015; Speers-Roesch et al., 2016). Glycogen is catabolized to form free glucose that, through glycolytic pathways, is employed to produce energy under stressful situations in cephalopods (Speers-Roesch et al., 2016). This situation is observed in Octopodidae species after capture by bottom-trawling, with muscle glycogen stores gradually being consumed to maintain circulating free glucose levels constant (presumably at homeostatic basal levels in these species). This carbohydrate consumption is evidenced during the first hours after stress, and reached metabolic equilibrium after 6–24 h recovery in onboard tanks. In vertebrates, lactate dehydrogenase (LDH) activity under anaerobic conditions like those described in this study produced lactate as a final end product of glycolysis (Gladden, 2004). However, our results highlighted a negligible amount of lactate in the arm’s muscle of Octopodidae, and low and constant levels of circulating plasma lactate. This metabolic response may be related to the low LDH activity in *O. vulgaris* paralarvae (Morales et al., 2017), or the lack of LDH activity in *S. officinalis* (Speers-Roesch et al., 2016), resulting in a striking result that deserve future evaluation of anaerobic catabolism in cephalopods.

### Amino Acids Metabolism

It was stated that *O. vulgaris* and other cephalopods have a protein-dominated metabolism (Katsanevakis et al., 2005; Petza et al., 2006), which explains the great amount of plasma and muscle amino acids in all three species in this study after an acute-stress situation. Free amino acids catabolism in octopus indicates a well-adapted use of proteins as a source of energy (Roumbedakis et al., 2017), and are employed as gluconeogenic substrates that are incorporated into glycogen deposits (Hochachka and Fields, 1982) and plasma glucose (Linares et al., 2015). In other invertebrates such as the oyster *Crassostrea virginica*, relying on amino acids catabolism to face anoxia also requires some glycolytic energy production to meet overall tissue requirements of recovery, returning to control values by 24 h, as described in the present study in all three Octopodidae species (Eberlee et al., 1983). Further studies describing the consumption of specific essential and non-essential amino acids during acute-stress situations will be of help for the aquaculture industry, as the development of specific diets containing those amino acids may minimize negative consequences of stress.

## Conclusion

This study described, for the first time, physiological acute-stress responses in three Octopodidae species. Recovery processes include maintaining the acid-base balance of the blood, and returning to basal levels of immune system parameters (such as hemocyanin, lysozyme, and peroxidase activities), as well as mobilization of energy molecules such as carbohydrates and amino acids to face the stressful process. Designed as a pilot study onboard a fishing vessel, further approaches should be conducted in controlled lab-conditions to better characterize secondary stress responses in cephalopods. Future studies could also measure physiological parameters *in vivo* at fixed time points, to reveal the exact time-course of plasma biomarkers after a stress situation. Aquaculture of these species may guarantee proper welfare conditions for their culture, but nowadays the lack of knowledge about the stress responses of these animals, among other issues, slows the development of this industry worldwide.

## Ethics Statement

Animals were kept and handled following the guidelines for experimental procedures in animal research from the Ethics and Animal Welfare Committee of the University of Cadiz, according to the Spanish (RD53/2013) and European Union (2010/63/UE) legislation. All experiments have been carried out under a special permit of scientific fishing granted to the Spanish Institute of Oceanography, and approved by the Spanish General Secretariat of Fisheries (project SUREDEPAR, Fundación Biodiversidad, Ministry for the Ecological Transition, Spain).

## Author Contributions

CB-M, IS, BC, JM, and IR-J conceived and designed the study. CB-M, AM-R, SF-B, and IR-J carried out experimental procedures. CB-M, AM-R, SF-B, BC, and IR-J analyzed and interpreted the data. CB-M, IS, BC, JM, and IR-J wrote the original draft. All authors reviewed, edited, and approved the final version of the manuscript.

## Conflict of Interest Statement

The authors declare that the research was conducted in the absence of any commercial or financial relationships that could be construed as a potential conflict of interest.

## References

[B1] Adachi K. Hirata T. Nishioka T. Sakaguchi M. (2003). Hemocyte components in crustaceans convert hemocyanin into a phenoloxidase-like enzyme. *Comp. Biochem. Physiol. B Biochem. Mol. Biol.* 134 135–141. 10.1016/s1096-4959(02)00220-8 12524041

[B2] Aguila J. Cuzon G. Pascual C. Domingues P. M. Gaxiola G. Sanchez A. (2007). The effects of fish hydrolysate (P) level on *Octopus maya* (Voss and Solis) diet: digestive enzyme activity, blood metabolites, and energy balance. *Aquaculture* 273 641–655. 10.1016/j.aquaculture.2007.07.010

[B3] ANON (2015). *Manual for the International Bottom Trawl Surveys Revision IX.* Copenhagen: ICES.

[B4] Arkhipkin A. I. Bizikov V. A. Doubleday Z. A. Laptikhovsky V. V. Lischchenko F. V. Perales-Raya C. (2018). Techniques for Estimating the Age and Growth of Molluscs: *Cephalopoda*. *J. Shellfish Res.* 37 783–792. 10.2983/035.037.0409

[B5] Barragán-Méndez C. Ruiz-Jarabo I. Fuentes J. Mancera J. M. Sobrino I. (2019). Survival rates and physiological recovery responses in the lesser-spotted catshark (*Scyliorhinus canicula*) after bottom-trawling. *Comp. Biochem. Physiol. A* 233 1–9. 10.1016/j.cbpa.2019.03.016 30905654

[B6] Barragán-Méndez C. Sánchez-García F. Sobrino I. Mancera J. M. Ruiz-Jarabo I. (2018). Air exposure in catshark (*Scyliorhinus canicula*) modify muscle texture properties: a pilot study. *Fishes* 3 1–11.29683143

[B7] Barton B. A. (2002). Stress in fishes: a diversity of responses with particular reference to changes in circulating corticosteroids. *Integr. Comp. Biol.* 42 517–525. 10.1093/icb/42.3.517 21708747

[B8] Bergmann M. Taylor A. C. Geoffrey Moore P. (2001). Physiological stress in decapod crustaceans (*Munida rugosa* and *Liocarcinus depurator*) discarded in the Clyde *Nephrops* fishery. *J. Exp. Marine Biol. Ecol.* 259 215–229. 10.1016/s0022-0981(01)00231-3 11343713

[B9] Boutilier R. G. Iwama G. K. Heming T. A. Randall D. J. (1985). The apparent pK of carbonic acid in rainbow trout blood plasma between 5 and 15 degrees C. *Respir. Physiol.* 61 237–254. 10.1016/0034-5687(85)90129-x 3931193

[B10] Castellanos-Martinez S. Diz A. P. Alvarez-Chaver P. Gestal C. (2014). Proteomic characterization of the hemolymph of *Octopus vulgaris* infected by the protozoan parasite *Aggregata octopiana*. *J. Proteomics* 105 151–163. 10.1016/j.jprot.2013.12.008 24370682

[B11] Chrousos G. P. (2009). Stress and disorders of the stress system. *Nat. Rev. Endocrinol.* 5 374–381. 10.1038/nrendo.2009.106 19488073

[B12] Clements J. C. Davidson J. D. P. Mcquillan J. G. Comeau L. A. (2018). Increased mortality of harvested eastern oysters (*Crassostrea virginaca*) is associated with air exposure and temperature during a spring fishery in Atlantic Canada. *Fish. Res.* 206 27–34. 10.1016/j.fishres.2018.04.022

[B13] Conners M. E. Levine M. (2017). Characteristics and discard mortality of octopus bycatch in Alaska groundfish fisheries. *Fish. Res.* 185 169–175. 10.1016/j.fishres.2016.09.010

[B14] Costas B. Conceicao L. Aragao C. Martos J. A. Ruiz-Jarabo I. Mancera J. (2011). Physiological responses of Senegalese sole (*Solea senegalensis* Kaup, 1858) after stress challenge: effects on non-specific immune parameters, plasma free amino acids and energy metabolism. *Aquaculture* 316 68–76. 10.1016/j.aquaculture.2011.03.011

[B15] Decker H. Jaenicke E. (2004). Recent findings on phenoloxidase activity and antimicrobial activity of hemocyanins. *Dev. Comp. Immunol.* 28 673–687. 10.1016/j.dci.2003.11.007 15043938

[B16] Eberlee J. C. Storey J. M. Storey K. B. (1983). Anaerobiosis, recovery from anoxia, and the role of strombine and alanopine in the oyster *Crassostrea virginica*. *Can. J. Zool.* 61 2682–2687. 10.1139/z83-353

[B17] Ellis A. E. (1990). “Lysozyme assays,” in *Techniques in Fish Immunology*, eds Stolen J. S. Fletcher T. C. Anderson D. P. Roberson B. Van Muiswinkel W. B. (Fair Haven: SOS Publications), 101–103.

[B18] Estefanell J. Socorro J. Afonso J. M. Roo J. Hernández-Palacios H. Izquierdo M. S. (2011). Evaluation of two anaesthetic agents and the passive integrated transponder tagging system in *Octopus vulgaris* (Cuvier 1797). *Aqua. Res.* 42 399–406. 10.1111/j.1365-2109.2010.02634.x

[B19] FAO (2018). *The State of World Fisheries and Aquaculture 2018.* Rome: FAO.

[B20] Gallagher A. J. Serafy J. E. Cooke S. J. Hammerschlag N. (2014). Physiological stress response, reflex impairment, and survival of five sympatric shark species following experimental capture and release. *Marine Ecol. Progr. Series* 496 207–218. 10.3354/meps10490

[B21] Garrido D. Varó I. Morales A. E. Hidalgo M. C. Navarro J. C. Hontoria F. (2017). Assessment of stress and nutritional biomarkers in cultured *Octopus vulgaris* paralarvae: effects of geographical origin and dietary regime. *Aquaculture* 468 558–568. 10.1016/j.aquaculture.2016.11.023

[B22] Gladden L. B. (2004). Lactate metabolism: a new paradigm for the third millennium. *J. Physiol.* 558 5–30. 10.1113/jphysiol.2003.058701 15131240 PMC1664920

[B23] Guttman O. Baranovski B. M. Schuster R. Kaner Z. Freixo-Lima G. S. Bahar N. (2015). Acute-phase protein alpha1-anti-trypsin: diverting injurious innate and adaptive immune responses from non-authentic threats. *Clin. Exp. Immunol.* 179 161–172. 10.1111/cei.12476 25351931 PMC4298394

[B24] Hochachka P. W. Fields J. H. A. (1982). Arginine, glutamine, and proline as substrates for oxidation and or glycogenesis in cephalopod tissues. *Pacific Sci.* 36 325–335. 17911304

[B25] Hochachka P. W. Fields J. H. A. Mommsen T. P. (1983). “Metabolic and enzyme regulation during rest-to-work transition: a mammal versus mollusc comparison,” in *Metabolic Biochemistry and Molecular Biomechanics*, eds Hochachka P. W. Wilbur K. M. (New York, NY: Academic Press), 67–90.

[B26] Howell B. J. Rahn H. Goodfellow D. Herreid C. (1973). Acid-Base regulation and temperature in selected invertebrates as a function of temperature. *Am. Zool.* 13 557–563. 10.1093/icb/13.2.557

[B27] Jereb P. Allcock A. L. Lefkaditou E. Piatkowski U. Hastie L. C. Pierce G. J. (2015). *Cephalopod biology and fisheries in Europe: II. Species Accounts.* Copenhagen: ICES International Council for the Exploration of the Sea.

[B28] Ji P. F. Yao C. L. Wang Z. Y. (2009). Immune response and gene expression in shrimp (*Litopenaeus vannamei*) hemocytes and hepatopancreas against some pathogen-associated molecular patterns. *Fish Shellfish Immunol.* 27 563–570. 10.1016/j.fsi.2009.08.001 19683058

[B29] Katsanevakis S. Stephanopoulou S. Miliou H. Moraitou-Apostolopoulou M. Verriopoulos G. (2005). Oxygen consumption and ammonia excretion of *Octopus vulgaris* (*Cephalopoda*) in relation to body mass and temperature. *Marine Biol.* 146 725–732. 10.1007/s00227-004-1473-9

[B30] Keppler D. Decker K. (1974). “Glycogen determination with amyloglucosidase,” in *Methods of Enzymatic Analysis*, ed. Bergmeyer H. U. (New York, NY: Academic Press), 1127–1131.

[B31] Kowaltowski A. J. De Souza-Pinto N. C. Castilho R. F. Vercesi A. E. (2009). Mitochondria and reactive oxygen species. *Free Radic. Biol. Med.* 47 333–343. 19427899 10.1016/j.freeradbiomed.2009.05.004

[B32] Lacoue-Labarthe T. Bustamante P. Horlin E. Luna-Acosta A. Bado-Nilles A. Thomas-Guyon H. (2009). Phenoloxidase activation in the embryo of the common cuttlefish *Sepia officinalis* and responses to the Ag and Cu exposure. *Fish Shellfish Immunol.* 27 516–521. 10.1016/j.fsi.2009.07.002 19616632

[B33] Lamarre S. G. Maccormack T. J. Sykes A. V. Hall J. R. Speers-Roesch B. Callaghan N. I. (2016). Metabolic rate and rates of protein turnover in food-deprived cuttlefish, *Sepia officinalis* (Linnaeus 1758). *Am. J. Physiol. Regulat. Integr. Comp. Physiol.* 310 R1160–R1168. 10.1152/ajpregu.00459.2015 27053650 PMC4935498

[B34] Lawrence M. J. Jain-Schlaepfer S. Zolderdo A. J. Algera D. A. Gilour K. M. Gallagher A. J. (2018). Are 3-minutes good enough for obtaining baseline physiological samples from teleost fish? *Can. J. Zool.* 96 774–786. 10.1139/cjz-2017-0093

[B35] Lee Y. J. Choi K. S. Lee D. S. Lee W. C. Park H. J. Choy E. J. (2015). The role of the adductor muscle as an energy storage organ in the pen shell *Atrina japonica* (Reeve, 1858). *J. Molluscan Studies* 81 502–511. 10.1093/mollus/eyv025

[B36] Linares M. Rodríguez S. Caamal-Monsreal C. Olivares A. Zuñiga O. Sanchez A. (2015). Timing of digestion, absorption and assimilation of octopus species living in tropical (*Octopus maya*) and subtropical-temperate (O. mimus) ecosystems. *Aqua. Biol.* 24 127–140. 10.3354/ab00642

[B37] Lund H. S. Wang T. Chang E. S. Pedersen L. F. Taylor E. W. Pedersen P. B. (2009). Recovery by the Norway lobster *Nephrops norvegicus* (L.) from the physiological stresses of trawling: influence of season and live-storage position. *J. Exp. Marine Biol. Ecol.* 373 124–132. 10.1016/j.jembe.2009.04.004

[B38] Machado M. Azeredo R. Diaz-Rosales P. Afonso A. Peres H. Oliva-Teles A. (2015). Dietary tryptophan and methionine as modulators of European seabass (*Dicentrarchus labrax*) immune status and inflammatory response. *Fish Shellfish Immunol.* 42 353–362. 10.1016/j.fsi.2014.11.024 25463296

[B39] Malham S. K. Coulson C. L. Runham N. W. (1998a). Effects of repeated sampling on the haemocytes and haemolymph of *Eledone cirrhosa* (Lam.). *Comp. Biochem. Physiol. Mol. Integr. Physiol.* 121 431–440. 10.1016/s1095-6433(98)10154-x

[B40] Malham S. K. Runham N. W. Secombes C. J. (1998b). Lysozyme and antiprotease activity in the lesser octopus *Eledone cirrhosa* (Lam.) (*Cephalopoda)*. *Dev. Comp. Immunol.* 22 27–37. 10.1016/s0145-305x(97)00045-1 9617581

[B41] Malham S. K. Lacoste A. Gelebart F. Cueff A. Poulet S. A. (2002). A first insight into stress-induced neuroendocrine and immune changes in the octopus *Eledone cirrhosa*. *Aqua. Living Res.* 15 187–192. 10.1016/s0990-7440(02)01173-7

[B42] Malham S. K. Runham N. W. (1998). A brief review of the immunology of *Eledone cirrhosa*. *South Af. J. Aqua. Sci.* 20 385–391.

[B43] Mandic M. Regan M. D. (2018). Can variation among hypoxic environments explain why different fish species use different hypoxic survival strategies? *J. Exp. Biol.* 221:jeb161349. 10.1242/jeb.161349 30381477

[B44] Martinez-Montaño E. Uriarte I. Rosas C. Amthauer R. Romero A. Farias A. (2017). Replacing live feed with formulated diets in juvenile Patagonian red octopus (*Enteroctopus megalocyathus*). *Aqua. Nutr.* 24:11 10.7773/cm.v37i1.1736

[B45] Mehault S. Morandeau F. Kopp D. (2016). Survival of discarded *Nephrops norvegicus* after trawling in the Bay of Biscay. *Fish. Res.* 183 396–400. 10.1016/j.fishres.2016.07.011

[B46] Moore S. (1968). Amino acid analysis: aqueous dimethyl sulfoxide as solvent for the ninhydrin reaction. *J. Biol. Chem.* 243 6281–6283. 5723468

[B47] Morales A. E. Cardenete G. Hidalgo M. C. Garrido D. Martin M. V. Almansa E. (2017). Time course of metabolic capacities in paralarvae of the common octopus, *Octopus vulgaris*, in the first stages of life. Searching biomarkers of nutritional imbalance. *Front. Physiol.* 8:427. 10.3389/fphys.2017.00427 28670288 PMC5473251

[B48] Mouritsen O. G. Styrbaek K. (2018). Cephalopod gastronomy - a promise for the future. *Front. Comm.* 3:38 10.3389/fcomm.2018.00038

[B49] Nande M. Domingues P. Rosas C. (2018). Effects of temperature on the embryonic development of *Octopus vulgaris*. *J. Shellfish Res.* 37 1013–1020. 10.1007/s00360-013-0783-y 24100467

[B50] Oellermann M. Lieb B. Portner H. O. Semmens J. M. Mark F. C. (2015a). Blue blood on ice: modulated blood oxygen transport facilitates cold compensation and eurythermy in an Antarctic octopod. *Front. Zool.* 12:6. 10.1186/s12983-015-0097-x 25897316 PMC4403823

[B51] Oellermann M. Strugnell J. M. Lieb B. Mark F. C. (2015b). Positive selection in octopus haemocyanin indicates functional links to temperature adaptation. *BMC Evol. Biol.* 15:133. 10.1186/s12862-015-0411-4 26142723 PMC4491423

[B52] Otero J. Álvarez-Salgado X. A. González A. F. Souto C. Gilcoto M. Guerra A. (2016). Wind-driven upwelling effects on cephalopod paralarvae: *Octopus vulgaris* and Loliginidae off the Galician coast (NE Atlantic). *Progr. Oceanogr.* 141 130–413.

[B53] Pamenter M. E. Dzal Y. A. Thompson W. A. Milsom W. K. (2018). Do naked mole rats accumulate a metabolic acidosis or an oxygen debt in severe hypoxia? *J. Exp. Biol.* 2018:jeb.191197. 10.1242/jeb.191197 30573665

[B54] Petza D. Katsanevakis S. Verriopoulos G. (2006). Experimental evaluation of the energy balance in *Octopus vulgaris*, fed ad libitum on a high-lipid diet. *Marine Biol.* 148 827–832. 10.1007/s00227-005-0129-8

[B55] Pierce G. J. Allcock L. Bruno I. Bustamante P. González A. Guerra A. (2010). *Cephalopod Biology and Fisheries in Europe.* Copenhagen: ICES (International Council for the Exploration of the Sea).

[B56] Quade M. J. Roth J. A. (1997). A rapid, direct assay to measure degranulation of bovine neutrophil primary granules. *Vet. Immunol. Immunopathol.* 58 239–248. 10.1016/s0165-2427(97)00048-2 9436268

[B57] Raicevich S. Minute F. Finoia M. G. Caranfa F. Di Muro P. Scapolan L. (2014). Synergistic and antagonistic effects of thermal shock, air exposure, and fishing capture on the physiological stress of *Squilla mantis* (*Stomatopod*a). *PLoS One* 9:e105060. 10.1371/journal.pone.0105060 25133593 PMC4136847

[B58] Reid S. G. Bernier N. J. Perry S. F. (1998). The adrenergic stress response in fish: control of catecholamine storage and release. *Comp. Biochem. Physiol. C* 120 1–27. 10.1016/s0742-8413(98)00037-1 9827012

[B59] Rodríguez A. Esteban M. A. Meseguer J. (2003). Phagocytosis and peroxidase release by seabream (*Sparus aurata* L.) *leucocytes in response to yeast cells*. *Anat. Record art A* 272 415–423. 10.1002/ar.a.10048 12704699

[B60] Ross N. W. Firth K. J. Wang A. Burka J. F. Johnson S. C. (2000). Changes in hydrolytic enzyme activities of naive Atlantic salmon *Salmo salar* skin mucus due to infection with the salmon louse *Lepeophtheirus salmonis* and cortisol implantation. *Dis. Aquat. Organ.* 41 43–51. 10.3354/dao041043 10907138

[B61] Roumbedakis K. Mascaro M. Martins M. L. Gallardo P. Rosas C. Pascual C. (2017). Health status of post-spawning *Octopus maya* (*Cephalopoda: Octopodidae*) females from Yucatan Peninsula. *Mexico. Hydrobiologia* 808 23–34. 10.1007/s10750-017-3340-y

[B62] Roura A. Amor M. González A. F. Guerra A. Barton E. D. Strugnel J. M. (2019). Oceanographic processes shape genetic signatures of planktonic cephalopod paralarvae in two upwelling regions. *Progr. n Oceanogr.* 170 11–27. 10.1016/j.pocean.2018.10.005

[B63] Sánchez P. Maynou F. Demestre M. (2004). Modelling catch, effort and price in a juvenile E. cirrhosa fishery over a 10-year period. *Fish. Res.* 68 319–327. 10.1016/j.fishres.2003.11.008

[B64] Sancho M. D. C. Valverde J. C. Sironi J. S. Garcia B. G. (2015). Is copper supplementation required in formulated feeds for *Octopus vulgaris* (Cuvier, 1797)? *J. Shellfish Res.* 34 473–480. 10.2983/035.034.0231

[B65] Schreck C. B. Tort L. Farrell A. P. Brauner C. (2016). *Biology of Stress in Fish.* Cambridge: Academic Press.

[B66] Senozan N. M. Avinc A. Unver Z. (1988). Hemocyanin levels in *Octopus vulgaris* and the cuttlefish *Sepia officinalis* from the Aegean Sea. *Comp. Biochem. Physiol. A* 91 581–585. 10.1016/0300-9629(88)90638-x

[B67] Skrzynska A. K. Maiorano E. Bastaroli M. Naderi F. Miguez J. M. Martinez-Rodriguez G. (2018). Impact of air exposure on vasotocinergic and isotocinergic systems in gilthead sea bream (*Sparus aurata*): new insights on fish stress response. *Front. Physiol.* 9:15. 10.3389/fphys.2018.00096 29487539 PMC5816901

[B68] Sobrino I. Juarez A. Rey J. Romero Z. Baro J. (2011). Description of the clay pot fishery in the Gulf of Cádiz (SW Spain) for *Octopus vulgaris*: selectivity and exploitation pattern. *Fish. Res.* 108 283–290. 10.1016/j.fishres.2010.12.022

[B69] Sobrino I. Silva L. Bellido J. M. Ramos F. (2002). Rainfall, river discharges and sea temperature as factors affecting abundance of two coastal benthic cephalopod species in the Gulf of Cádiz (SW Spain). *Bull. Marine Sci.* 71 851–865.

[B70] Speers-Roesch B. Callaghan N. I. Maccormack T. J. Lamarre S. G. Sykes A. V. Driedzic W. R. (2016). Enzymatic capacities of metabolic fuel use in cuttlefish (*Sepia officinalis*) and responses to food deprivation: insight into the metabolic organization and starvation survival strategy of cephalopods. *J. Comp. Physiol. B Biochem. System. Environ. Physiol.* 186 711–725. 10.1007/s00360-016-0991-3 27138338

[B71] Storey K. B. Storey J. M. (1983). “Carbohydrate metabolism in cephalopod molluscs,” in *Metabolic Biochemistry and Molecular Biomechanics*, eds Hochachka P. W. Wilbur K. M. (New York, NY: Academic Press), 91–136. 10.1016/b978-0-12-751401-7.50010-7

[B72] Swain P. Dash S. Sahoo P. K. Routray P. Sahoo S. K. Gupta S. D. (2007). Non-specific immune parameters of brood Indian major carp *Labeo rohita* and their seasonal variations. *Fish Shellfish Immunol.* 22 38–43. 10.1016/j.fsi.2006.03.010 16679030

[B73] Tresguerres M. Hamilton T. J. (2017). Acid–base physiology, neurobiology and behaviour in relation to CO2-induced ocean acidification. *J. Exp. Biol.* 220 2136–2148. 10.1242/jeb.144113 28615486

[B74] Veldhuizen L. J. L. Berentsen P. B. M. De Boer I. J. M. Van De Vis J. W. Bokkers E. A. M. (2018). Fish welfare in capture fisheries: a review of injuries and mortality. *Fish. Res.* 204 41–48. 10.1016/j.fishres.2018.02.001

[B75] Wedemeyer G. A. Barton B. A. Mcleay D. J. (1990). “Stress and acclimation,” in *Methods of Fish Biology*, eds Schreck C. B. Moyle P. B. (Bethesda MD: American Fisheries Society), 451–489.

